# A novel gain-of-function mutation in *sgk-1* partially suppresses mTORC2 defects

**DOI:** 10.17912/micropub.biology.001163

**Published:** 2024-09-30

**Authors:** David Cully, Natalie R. Cohen, Peter C. Breen, Martin A. Newman, Robert H. Dowen

**Affiliations:** 1 Integrative Program for Biological and Genome Sciences, University of North Carolina at Chapel Hill, Chapel Hill, North Carolina, United States; 2 Department of Biology, University of North Carolina at Chapel Hill, Chapel Hill, North Carolina, United States; 3 Department of Cell Biology and Physiology, University of North Carolina at Chapel Hill, Chapel Hill, North Carolina, United States

## Abstract

The serine/threonine protein kinase SGK-1 is a downstream target of mTOR complex 2 (mTORC2) and is a conserved regulator of growth and metabolism. In *C. elegans* , mutations in *rict-1* , which encodes an essential component of mTORC2, impairs lipid homeostasis and growth; however, these defects are partially suppressed by an activating mutation in SGK-1 , E116K. Here, we describe a stronger gain-of-function mutation in *sgk-1* , L112F, that was identified in a forward genetic screen for *rict-1* suppressor mutations *.* This allele will be useful in further dissecting the mTORC2 pathway and provides new insight into the role of this conserved residue in regulating SGK-1 kinase activity.

**Figure 1. The sgk-1(rhd168[L112F]) gain-of-function allele is a genetic suppressor of the rict-1(mg360) mutation in C. elegans:**
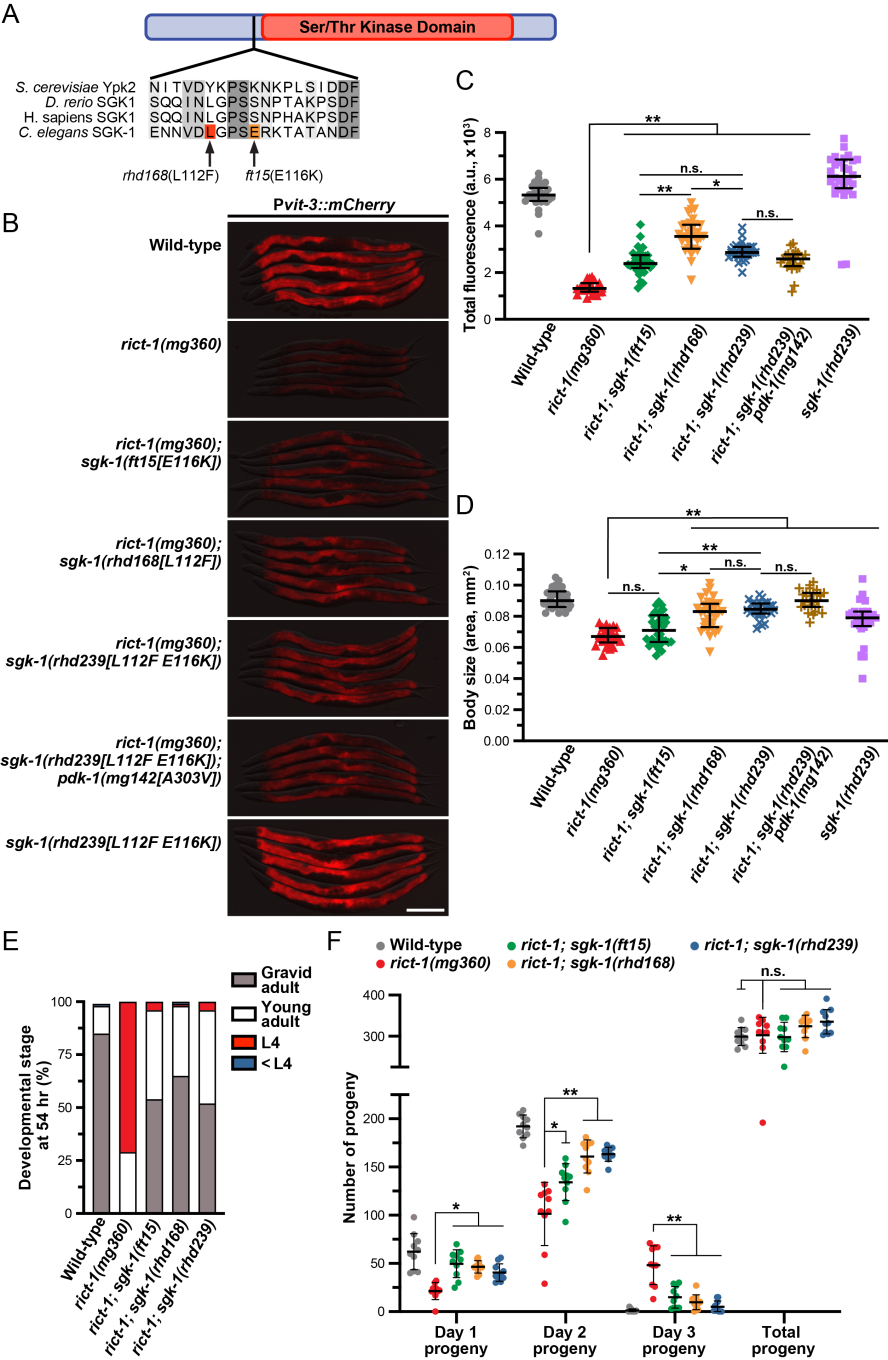
**A)** The *sgk-1 ( rhd168 )* allele, which is a missense mutation resulting in a L112F amino acid change, was isolated in a F1 suppressor screen using the *rict-1 ( mg360 );* P *vit-3 ::mCherry* strain. The L112F mutation is proximal to the previously described E116K gain-of-function mutation and is located just outside of the kinase domain. **B)** Representative overlaid DIC and mCherry fluorescence images (scale bar, 200 μm) and **C)** fluorescence quantification (median and interquartile range; n.s., not significant, *, *P* <0.002, **, *P* <0.0001, one-way ANOVA) of day 1 adult wild-type and mutant animals expressing the P *vit-3 ::mCherry* vitellogenesis reporter. Fluorescence in wild-type animals was significantly different from all other strains ( *P* <0.001). The *rhd168* allele restored mCherry expression to a greater extent than the *ft15* allele in the *rict-1 ( mg360 )* mutant background; however, combining the *rhd168* allele with other activating mutations did not further suppress the *rict-1* vitellogenesis defects. **D)** A body size analysis of day 1 adult wild-type and *rict-1* mutants carrying the indicated suppressor mutations (median and interquartile range; n.s., not significant, *, *P* =0.0004, **, *P* <0.0001, one-way ANOVA). The *rhd168* allele, but not the *ft15* allele, partially suppressed the small body size of the *rict-1* mutant. The body size of *rict-1 ( mg360 ); sgk-1 ( rhd239 )* animals was not significantly different from wild-type (one-way ANOVA). **E)** Developmental stages of wild-type and the indicated mutant animals after 54 hours of growth from the L1 stage at 20°C (n>120 for each genotype). The slow growth conferred by loss of *rict-1* is partially suppressed by all the *sgk-1* gain-of-function mutations to similar levels. **F)** Progeny production at day 1 (0-24 hr post L4), day 2 (25-48 hr post L4), and day 3 (49-72 hr post L4) of reproduction (total brood values are also shown; n=10 individuals per genotype; mean ± SD). The *rict-1* mutants display delayed reproductive output, which is partially suppressed by the *sgk-1* gain-of-function mutations (n.s., not significant, *, *P* <0.005, **, *P* <0.0001, one-way ANOVA). **B-F)** All strains contain the *rhdSi42* transgene.

## Description

The mechanistic target of rapamycin (mTOR) is an evolutionarily conserved serine-threonine protein kinase that responds to a wide array of nutritional inputs and growth factors to regulate cell growth and proliferation through activation of anabolic processes ( *i.e.* , protein, lipid, and nucleotide synthesis), modulation of metabolic gene expression programs, and inhibition of autophagy (Schmelzle and Hall 2000) . mTOR exists in at least two protein complexes, mTORC1 and mTORC2, and while mTORC1 has been studied extensively, much less is known about the mechanism of mTORC2 activation and the cellular processes that are under its control. Several AGC kinases (named after the protein kinase A, G, and C families) are well-established direct targets of mTORC2, including AKT/PKB (Ak strain transforming/protein kinase B), SGK (serum and glucocorticoid-regulated kinase), and PKC (protein kinase C), which together promote growth, cell survival, cell cycle progression, and anabolic metabolism in response to hormonal signals and subsequent mTORC2 activation (Sarbassov et al. 2004; Sarbassov et al. 2005; García-Martínez and Alessi 2008; Ikenoue et al. 2008) . Dysregulation of mTORC2 can alter glucose, amino acid, nucleotide, and lipid metabolism pathways to support tumorigenesis in several cancer types (Masui et al. 2014) .

SGK-1 , the *C. elegans* orthologue of the mammalian serum and glucocorticoid-inducible kinase, acts downstream of mTORC2 to promote growth, maintain lipid homeostasis, and support longevity through the regulation of autophagy (Soukas et al. 2009; Dowen et al. 2016; Aspernig et al. 2019; Zhou et al. 2019) . In *C. elegans* , mutation of *rict-1* , which encodes the mTORC2-specific scaffolding protein Rictor, produces similar phenotypes as loss of *sgk-1* , including impaired lipid homeostasis, reduced growth rate, induction of autophagy, and reduced lifespan (Jones et al. 2009; Soukas et al. 2009; Chen et al. 2013; Mizunuma et al. 2014; Dowen et al. 2016; Zhou et al. 2019) . We previously found that mutation of either *sgk-1* or *rict-1* reduces the expression of intestinal vitellogenin lipoproteins, which mediate the intestine-to-germline transport of lipids during reproduction (Dowen et al. 2016) . This reallocation of crucial metabolic resources is dynamically regulated by both developmental and nutritional inputs (Perez and Lehner 2019) .

We have previously employed forward genetic screens to identify proteins that regulate the expression of the vitellogenin genes ( *vit-1-6* ). Here, we leverage a transgenic strain carrying a single-copy *vit-3* transcriptional reporter, which expresses *mCherry* under the control of the *vit-3* promoter (P *vit-3 ::mCherry* ), to screen for mutations that suppress the vitellogenin expression defects observed in the *rict-1 ( mg360 )* mutant. Following EMS mutagenesis, we screened for *rict-1* suppressor mutants that displayed increased expression of the P *vit-3 ::mCherry* reporter. We specifically screened the F1 generation to enrich for gain-of-function mutations, which are likely to activate downstream signaling components, including SGK-1 . Of the five suppressor mutants that we isolated in the screen, only one, *rhd168* , had a clear lesion in the *sgk-1* gene based on Sanger sequencing of the genomic locus.

The putative gain-of-function *sgk-1 ( rhd168 )* mutation results in a L112F amino acid change proximal to the Ser/Thr kinase domain of SGK-1 ( Figure 1A ). Intriguingly, this mutation sits four residues upstream of the previously identified *sgk-1 ( ft15 [E116K])* gain-of-function mutation, which suppresses the developmental defects associated with the *rict-1 ( mg360 )* mutation (Jones et al. 2009) . Thus, we compared the ability of these two *sgk-1* gain-of-function mutations to suppress the *rict-1 ( mg360 )* vitellogenesis defects, finding that the *sgk-1 ( rhd168 [L112F])* allele was a stronger suppressor of the P *vit-3 ::mCherry* expression defects ( Figure 1B -C). Next, we engineered both mutations into the *sgk-1* locus using CRISPR/Cas9 genomic editing to assess whether these mutations would have an additive effect when introduced together; however, the double mutant allele, *sgk-1 ( rhd239 [L112F E116K])* , was slightly less effective than the *rhd168* allele in its ability to suppress the *rict-1 ( mg360 )* vitellogenesis defects. Intriguingly, the suppressive effects of the *rhd239 [L112F E116K]* allele on P *vit-3 ::mCherry* expression more closely resembled the effects of the *ft15 [E116K]* allele rather than the *rhd168 [L112F]* mutation ( Figure 1C ), suggesting that these two mutations may uniquely impact how SGK-1 engages and phosphorylates its downstream substrates, a difference that we are able to resolve using this highly sensitive reporter. Given that loss of *rict-1* reduces adult body size (Jones et al. 2009; Soukas et al. 2009) , we also tested whether each gain-of-function allele, as well as the combination of both mutations, could suppress the body size defects of the *rict-1 ( mg360 )* mutant. Consistent with the vitellogenesis phenotype, the *rhd168* allele was a stronger suppressor than the *ft15* allele and combining both mutations yielded no further effect ( Figure 1D ). Importantly, neither gain-of-function allele fully restored P *vit-3 ::mCherry* expression or body size to wild-type levels, suggesting that other factors could function downstream of mTORC2 to regulate vitellogenesis and growth. Consistent with this hypothesis, none of the *sgk-1* gain-of-function alleles reproducibly suppressed the lipid storage defects conferred by the *rict-1 ( mg360 )* mutation.

Alternatively, the gain-of-function mutations may only partially activate SGK-1 kinase activity and other factors and/or signaling pathways may be necessary to fully activate the kinase. In addition to phosphorylation of Ser ^422^ by mTORC2 in the hydrophobic motif, SGK1 is also phosphorylated by PDPK1 (also known as PDK1, phosphoinositide-dependent kinase 1) within the activation loop at Thr ^256^ and phosphorylation of both sites is required to confer maximal SGK1 activity in mammals (Kobayashi and Cohen 1999; García-Martínez and Alessi 2008) . It is presumed that phosphorylation of SGK-1 by mTORC2 and PDK-1 occurs at these same conserved sites in *C. elegans* . Thus, we crossed the previously characterized *pdk-1 ( mg142 )* gain-of-function mutation into the *rict-1 ( mg360 ); sgk-1 ( rhd239 [L112F E116K])* mutant background and measured P *vit-3 ::mCherry* expression and body size (Paradis et al. 1999) . The activating *pdk-1* mutation did not further suppress the vitellogenesis and body size defects observed in the *rict-1 ( mg360 )* mutant ( Figure 1B -D), suggesting that PDK-1 may not function in concert with mTORC2/ SGK-1 to regulate these physiological processes. Importantly, we cannot rule out the possibility that the effects conferred by the *pdk-1 ( mg142 )* mutation are specific to AKT signaling and/or the phenotype for which it was isolated ( *i.e.* , suppression of the *age-1 ( mg44 )* Daf-c phenotype) (Paradis et al. 1999) .

Impaired mTORC2/ SGK-1 signaling results in developmental delay and defects in progeny production (Jones et al. 2009; Soukas et al. 2009; Wang et al. 2014) . To assess whether the *sgk-1* gain-of-function mutations can suppress these defects, we first measured the developmental rate of wild-type and *rict-1 ( mg360 )* mutant animals at 54 hours post-L1 stage. Indeed, loss of *rict-1* slowed larval development; however, all three *sgk-1* gain-of-function mutations suppressed this developmental defect to near wild-type levels ( Figure 1E ). Consistent with this observation, the *rict-1 ( mg360 )* mutation conferred a delayed reproductive output ( Figure 1F ). This is congruent with the delayed reproduction displayed by *sgk-1* RNAi knockdown animals (Wang et al. 2014) . Critically, all three *sgk-1* gain-of-function mutations similarly suppressed the delay in reproduction caused by loss of *rict-1* ( Figure 1F ), which correlates with the levels of vitellogenin production in these different mutant backgrounds. Notably, we did not find that the total brood size was reduced in the *rict-1* mutant, which is inconsistent with previous observations (Soukas et al. 2009) . It is possible that different culturing conditions contribute to these discrepancies. Together, these data demonstrate that developmental rate and reproductive maturity are delayed in the *rict-1* mutant, which can be strongly suppressed by activating mutations in *sgk-1* .

In conclusion, our results indicate that the *sgk-1 ( rhd168 )* gain-of-function mutation is a stronger suppressor of the *rict-1 ( mg360 )* vitellogenesis and growth phenotypes than the existing *sgk-1 ( ft15 )* allele. Future studies will be needed to assess whether *sgk-1 ( rhd168 )* similarly suppresses other *rict-1* mutant phenotypes (Chen et al. 2013; Mizunuma et al. 2014) . It is intriguing that the L112F mutation sits at a conserved residue in SGK-1 (Leu ^85^ in humans) and that it is positioned proximal to the E116K mutation. Interestingly, a crystal structure of inactive human SGK1 revealed that Leu ^85^ occupies the N-domain hydrophobic pocket (Zhao et al. 2007) , which has been shown to dock the conserved FXXF hydrophobic motif in other AGC kinase family members (Knighton et al. 1991; Yang et al. 2002) . It is possible that Leu ^85^ stabilizes an inactive form of SGK1 or that it competes with the C-terminal FXXF hydrophobic motif for access of the N-domain hydrophobic pocket. These structural features could be altered by the L112F mutation. Given the proximity to Leu ^112^ , it is likely that the E116K mutation confers a structural change in the N-domain hydrophobic pocket of *C. elegans* SGK-1 that is similar to that produced by the L112F mutation; however, we predict that the L112F mutation is more effective in stabilizing the active form of the kinase, possibly due to structural constraints caused by the phenylalanine side chain. This is consistent with our observation that the L112F E116K double mutant more closely resembles the L112F single mutant in multiple assays ( Figure 1D, F ). Our study provides the framework to further investigate the role of this disordered region in human SGK1 (residues 66-92), which currently has no known function.

## Methods

*C. elegans strains and maintenance*

Animals were maintained at 20°C on Nematode Growth Media (NGM) plates seeded with *E. coli* OP50 as previously described (Brenner 1974) . The vitellogenesis reporter strain DLS537 was generated by fusing the *vit-3* promoter (518bp upstream of the start codon) to *mCherry:: unc-54 3'UTR* and inserted into the MosSCI-compatible pCFJ151 plasmid via Gibson assembly (Gibson et al. 2009) . The resulting plasmid was microinjected into *unc-119( ed3 )* animals to generate the single-copy integrant *rhdSi42 [* P *vit-3 ::mCherry:: unc-54 3'UTR]* via Mos1 transposition as previously described (Frøkjær-Jensen et al. 2008). The resulting transgenic strain was backcrossed to N2 wild-type three times to give DLS537 . The *rhdSi42* reporter was introduced into various mutant backgrounds by standard genetic crossing and the resulting strains were imaged on a Nikon SMZ-18 stereo microscope equipped with a DS-Qi2 monochrome camera.

*EMS mutagenesis*

The DLS547 strain was mutagenized with ethyl methanesulfonate (Sigma-Aldrich) as previously described (Dowen et al. 2016) . Approximately 50,000 synchronized F1 animals were screened for suppression of the P *vit-3 ::mCherry* expression defects that are associated with the *rict-1 ( mg360 )* mutation. The suppressor mutants were rescreened on *rict-1* RNAi (Dowen et al. 2016) to select against mutants that carry intragenic *rict-1* suppressor mutations. To identify causative mutations within the *sgk-1* locus, exons 2-4 and 5-8 were individually amplified by PCR from genomic DNA and subjected to Sanger sequencing. The strain carrying the *rhd168* allele was backcrossed to DLS547 four times to yield DLS575 .

*CRISPR/Cas9 gene editing*

To generate the *sgk-1 ( rhd239 [L112F E116K])* mutant we performed CRISPR/Cas9 gene editing to introduce the *E116K* mutation into the *sgk-1 ( rhd168 [L112F])* allele as previously described (Ghanta and Mello 2020) . The crRNA sequence was 5'-UUGAUUUCGGACCAAGUGAGGUUUUAGAGCUAUGCU-3' and the ssODN repair sequence was 5'-AATCCTTATGGCCAAAACATACGTTTTTCTCTTACTTGGTCCGAAATCAACATTATTCTC-3' (Integrated DNA Technologies). The Cas9::crRNA:tracrRNA complexes along with the ssODN repair template were microinjected into the DLS575 strain to generate DLS704 . DLS698 was generated by crossing DLS704 to DLS537 to remove the *rict-1 ( mg360 )* mutation.

*Vitellogenesis reporter analysis*

To perform a qualitative analysis of P *vit-3 ::mCherry* reporter expression, L4 animals were transferred to new plates and allowed to develop for 24 hr (day 1 adults). Five animals from each strain were immobilized on a 2% agarose pad with 25mM levamisole. All brightfield (10 ms exposure) and mCherry (300 ms exposure) images were taken at 10X magnification.

*Body size and vitellogenesis reporter quantification*

Forty L4 individuals from each strain were transferred to new plates and grown for 24 hr as described above. Brightfield and mCherry fluorescence (300ms) images were taken of individual worms at 10X magnification. Each animal in the brightfield images was outlined using the freehand drawing tool in the image-processing software ImageJ v1.53 (Schindelin et al. 2012) . Body size was measured as the two-dimensional area (in mm ^2^ ) and the data for each strain are presented as the median and interquartile range. Similarly, P *vit-3 ::mCherry* fluorescence was quantified by outlining the intestinal cells and then calculating the mean intensity (gray value) within this region using ImageJ v1.53. The data were plotted in Prism 10 as the median and interquartile range. For both analyses, a one-way ANOVA with a Bonferroni correction for multiple testing was performed.

*Growth rate assay*

Embryos were isolated from gravid adults by bleaching and were incubated overnight at room temperature in M9 buffer with rotation, resulting in a population of synchronized L1 animals. Approximately 125 L1s per genotype were dropped on NGM plates and grown for 54 hours. Animals were scored as adults, young adults, or L4s based on their vulva morphology and whether they possessed embryos in their uterus. The experiment was performed twice with identical results.

*Brood Size*

Animals were grown for at least two generations under well-fed conditions before L4s were singled to individual plates (12 animals per genotype). The individuals were transferred to new plates every 24 hours for 5 days. The resulting progeny were allowed to develop to at least the L4 stage before they were counted. For each genotype, the individual with the highest and lowest total progeny production was dropped from the analysis. The counts for day 1, 2, and 3 of progeny production (and the total brood values) were plotted in Prism 10 as the mean ± the standard deviation and a one-way ANOVA with a Bonferroni correction for multiple testing was performed to determine statistical significance.

## Reagents

**Table:** 

**Strain**	**Genotype**	**Available From**
N2	wild-type	CGC
DLS537	*rhdSi42 [P vit-3 ::mCherry:: unc-54 3'UTR + cb-unc-119(+)] II *	Upon request
DLS547	*rhdSi42 [P vit-3 ::mCherry:: unc-54 3'UTR + cb-unc-119(+)] rict-1 ( mg360 ) II *	Upon request
DLS575	*rhdSi42 [P vit-3 ::mCherry:: unc-54 3'UTR + cb-unc-119(+)] rict-1 ( mg360 ) II; sgk-1 ( rhd168 [L112F]) X *	Upon request
DLS698	*rhdSi42 [P vit-3 ::mCherry:: unc-54 3'UTR + cb-unc-119(+)]; sgk-1 ( rhd239 [L112F, E116K]) X *	Upon request
DLS704	*rhdSi42 [P vit-3 ::mCherry:: unc-54 3'UTR + cb-unc-119(+)] rict-1 ( mg360 ) II; sgk-1 ( rhd239 [L112F, E116K]) X *	Upon request
DLS820	*rhdSi42 [P vit-3 ::mCherry:: unc-54 3'UTR + cb-unc-119(+)] rict-1 ( mg360 ) II; sgk-1 ( ft15 [E116K]) X *	Upon request
DLS821	*rhdSi42 [P vit-3 ::mCherry:: unc-54 3'UTR + cb-unc-119(+)] rict-1 ( mg360 ) II; pdk-1 ( mg142 ) sgk-1 ( rhd239 [L112F, E116K]) X *	Upon request
